# Ten outcome measures in forensic mental health: A survey of clinician views on comprehensiveness, ease of use and relevance

**DOI:** 10.1002/cbm.2221

**Published:** 2021-11-09

**Authors:** Howard Ryland, Jonathan Cook, Ray Fitzpatrick, Seena Fazel

**Affiliations:** ^1^ Department of Psychiatry University of Oxford Oxford UK; ^2^ Nuffield Department of Orthopaedics, Rheumatology and Musculoskeletal Sciences University of Oxford Oxford UK; ^3^ Nuffield Department of Population Health University of Oxford Oxford UK

**Keywords:** forensic psychiatry, outcome measurement, quality of life, risk assessment

## Abstract

**Background:**

Measurement of outcomes in forensic mental health services is essential to ensure that these services are delivering good quality care and treatment. Instruments for outcome measurement should cover all important domains, be easy to implement in a routine clinical context and facilitate transfer of relevant information between clinicians as the patient progresses along a recovery and rehabilitation pathway.

**Aims:**

We sought the views of clinicians on 10 common instruments used as outcome measures in forensic mental health services, especially on their perceived comprehensiveness and ease of use.

**Methods:**

An online survey was used to gather the views of clinicians from a range of professional backgrounds working in forensic mental health services in the United Kingdom. The selected instruments were identified from a previous systematic review of instruments for measuring outcomes in this context. Questions covered comprehensiveness, ease of use, patient involvement, relevance and use for progressing tracking and care planning.

**Results:**

Complete responses were received from 229 individuals. The range of respondents either agreeing or strongly agreeing that individual instruments were comprehensive was 6–39%; easy to use 19%–69%; relevant 31%–78%; useful to measure progress 7–70%; and useful for care planning 33–81%. Respondents reported that, for each of the 10 instruments, full involvement of patients varied between 3% and 22%; partial involvement 12–45%, patients informed, but not involved 11%–28%; and patients not involved or informed 21%‐64%.

**Conclusions:**

The Health of the Nation Outcome Scale Secure, the only instrument designed as an outcome measure, is not regarded by clinicians as useful in that respect and the majority of clinicians do not inform patients they are using it. Clinicians appear most familiar with the Historical Clinical Risk 20 (HCR‐20), which some respondents considered potentially useful as a progress measure but with limited patient involvement. Most respondents did not think that the HCR‐20 is comprehensive. There is a need for outcome measures that are comprehensive, easy to use and have adequate patient involvement in their development and rating.

## 1 INTRODUCTION

Forensic mental health services in high‐income countries are usually high‐cost, low‐volume services, with a high average length of stay. For example, in England in 2013, there were 2500 low secure, 2800 medium secure and 630 high secure beds, costing £153,300, £176,295 and £271,560–357,335 per bed, per year respectively (Centre for Mental Health, 2013; Huband et al., 2018; Kennedy, Simpson, & Haque, 2019). The average length of stay is long, with as many as 27% of patients staying over 10 years (Holley, Weaver, & Vollm, 2020). Specialist forensic inpatient capacity has increased in many Western countries since the 1990s, in contrast to the deinstitutionalisation seen in other areas of psychiatry (Jansman‐Hart et al., 2011). There is some evidence that patients discharged from such services have lower rates of repeat offending than comparable groups, but in other areas, such as suicide, poor outcomes are common (Fazel et al., 2016). Measurement of patient outcomes is necessary to ensure that forensic mental health services are effective in their given tasks, including public protection to ensure that patients do no significant harm, mental health improvement and psychosocial functional benefits for patients. Furthermore, it is important that resources are used most efficiently. Outcome measures can be used to track the progress of individual patients and provide insights into the functioning of a unit at a systemic level (Lewis & Killaspy, 2014). An understanding of progress through outcome measurement can help to identify outstanding areas of need and targets for future interventions (Simpson & Penney, 2011).

Governmental policies are increasingly concerned with measuring outcomes that matter to all relevant parties, including patients, clinicians, relatives, policy makers and justice ministries (NHS England and NHS Improvement, 2016; Tzelepis et al., 2015). Determining whether agreed outcomes occur requires instruments that capture the perspective of all key stakeholders. For clarity of representation, these usually take the form of standardised questionnaires or rating scales. Particularly as they carry some implication of scientific approach, it is essential that such outcome measures are fit for purpose. This includes ensuring that appropriately trained individuals are rating these tools and items reflect issues that are genuinely important for the various stakeholders. The content of instruments must cover all important outcome domains, including quality of life and functional outcomes as well as risk and clinical symptoms (Shinkfield & Ogloff, 2015). Given the increasing workload experienced by clinicians within forensic mental health services, outcome measures should be simple to understand and quick to use if they are to be effective in clinical practice (Berry & Robertson, 2019; Newman et al., 2020). Staff members completing such outcome measures should receive formal training in their administration (Hatfield & Ogles, 2007).

Previous systematic reviews have identified a large number of outcome measurement instruments used in forensic mental health services for both research and clinical purposes (Fitzpatrick, Chambers, et al., 2010; Shinkfield & Ogloff, 2015). A recent systematic review identified 435 different instruments, or their variants, used as outcome measures in forensic mental health services (Ryland et al., 2021). This included instruments that were primarily designed for another purpose, in particular predicting risk of harm to others and assessments of need, provided that they had at least some dynamic items which could be used to track progress over time. The majority of the instruments identified were clinician reported. As part of the review, a quality assessment was conducted of the top 10 most common instruments designed for use in a forensic setting and that included multiple domains. This was performed in line with an adaptation of the COnsensus‐based Standards for health Measurement INstruments (COSMIN) systematic review guidelines (Prinsen et al., 2018). The COSMIN methodology is a well‐recognised approach to evaluating outcome measures, which can be used to determine the quality of different types of measure, such as clinician and patient reported instruments (Mokkink et al., 2016).

The COSMIN guidelines emphasise the importance of evidence for good content validity, which includes comprehensiveness, relevance and comprehensibility (Terwee et al., 2018). Good content validity should result from input from an adequate range of people with a valid interest in the outcome(s). All but one of the top 10 instruments assessed by the review, the Camberwell Assessment of Need Forensic version (CANFOR), had inadequate evidence for content validity (Thomas et al., 2008).

Although the literature provides information on many psychometric properties, including some aspects of content validity, it is not informative in how measures are used in practice. Empirical evidence of practitioner appraisal of content validity and practical applicability of these instruments is therefore necessary to understand their relevance to, and feasibility of use within, routine clinical practice. Such evidence can be derived through a variety of approaches, including qualitative interviews or focus groups, and using survey methodology (Fitzpatrick, Davey, et al., 1998).

Our aim was to explore clinician‐perceived content validity and practical applicability of some of the most popular outcome measures used in forensic mental health services in the United Kingdom.

## 2 METHODS

### 2.1 Ethics approval

Ethical approval was granted by NHS London‐Surrey Research Ethics Committee, with reference 18/LO/0929.

### 2.2 Study design

This study was an internet‐based survey of clinicians working in forensic mental health services in the United Kingdom.

### 2.3 Participants

Any clinician working in forensic mental health services in the United Kingdom was eligible, regardless of professional background. People were invited to take part through a number of professional networks. Invitations containing a link to the survey online were distributed via emails and newsletters sent by the Royal College of Psychiatrists' [the College] Forensic Psychiatry Faculty, the Adult Secure Services Clinical Reference Group at NHS England, the Forensic Network of Scotland and the College Centre for Quality Improvement (CCQI) Quality Network for Forensic Mental Health Services. The CCQI oversees several multidisciplinary networks which set standards for a range of services across mental healthcare and then conduct peer review visits to evaluate individual providers against those standards. Further participants were recruited through snowball sampling by existing participants forwarding the invitation to their colleagues. Patients and carers were not approached for their views, as the survey required respondents to be familiar with the instruments, of which the majority were solely clinician‐reported.

### 2.4 Questionnaire development and data collection

The research team developed the survey, which was then piloted with a forensic psychiatrist from outside the research team and feedback incorporated before finalising the survey.

The 10 instruments chosen for clinician responding (see Table 1) were those identified through a systematic review of the literature, which followed PRISMA guidelines and reported separately, as those designed for use in forensic mental health settings, with multiple outcome domains, and appearing most commonly in the published literature (Ryland et al., 2021).

**TABLE 1:** Instruments included in the survey

Instrument	Brief description
HCR‐20 (Douglas & Belfrage, 2014)	SPJ risk assessment currently in its third version, which has one static and two dynamic clinician reported scales
START (Webster et al., 2006)	Used by clinicians to assess the strengths and vulnerabilities of forensic patients
CANFOR (Thomas et al., 2008)	Clinician and patient rated scales with parallel items to assess met and unmet needs
DUNDRUM (O'Dwyer et al., 2011)	Quartet of clinician reported scales with two further patient reported scales, designed to aid decision making regarding level of security for forensic patients
HoNOS Secure (Dickens et al., 2007)	Clinician reported progress measure with clinical and security scales
LS/CMI (Andrews et al., 2004).	Latest iteration in the level of service family of instruments, designed primarily for use in criminal justice settings
VRS (Wong & Gordon, 2006)	Combines static and dynamic factors to assess risk, readiness to change and identify targets for interventions
SAPROF (de Vogel et al., 2009)	Designed to complement traditional risk assessments by considering protective factors
SVR‐20 (Boer et al., 1997)	SPJ instrument designed to specifically assess the risk of sexual violence
BEST Index (Woods & Reed, 1999)	A nurse‐reported assessment of behaviour with six subscale

Abbreviations: BEST, Behavioural Status; CANFOR, Camberwell Assessment of Need—Forensic; DUNDRUM, Dangerousness, Understanding, Recovery and Urgency Manual; HCR‐20, The Historical Clinical Risk 20; HoNOS, Health of the Nation Outcome Scale; LS/CMI, Level of Service Case Management Inventory; SVR‐20, Sexual Violence Risk 20; START, Short Term Assessment of Risk and Treatability; SAPROF, Structured Assessment of Protective Factors for risk of violence; SPJ, structured professional judgement; VRS, Violence Risk Scale.

Respondents were asked for demographic information, including their geographical location and professional background. They were allowed to identify one or more setting that they worked in. Respondents rated their familiarity with the selected instruments and their level of training in using them. The survey operated so that if a participant had never heard of a particular instrument, they received no further questions about that instrument, in order to minimise participant burden. Respondents considered the level of patient involvement in rating these tools, ranging from patients being fully involved as equal partners, to patients not being informed of their ratings at all. Respondents were asked about the comprehensiveness and ease of use of the selected instruments. They were also able to comment on the relevance of outcome measure to forensic services, care planning and tracking patients' progress. Response options for the questions on content validity and practical applicability were five‐point Likert scales ranging from strongly agree to strongly disagree. Respondents could also add additional information in free text comments. The survey was operationalised using the Qualtrics survey platform which allowed users to respond online (Qualtrics, 2019). Data collection was between 8 October 2019 and 2 March 2020.

### 2.5 Data analysis

Descriptive statistics were used to characterise the sample in terms of geographical location, professional background and service in which they worked, and to analyse responses to the questions about familiarity with each scale, degree of training in its use and level of patient involvement. Responses concerning content validity and practical applicability were analysed according to the five point Likert scale. ‘Don't know’ responses to individual questions about instruments were assumed to indicate that the respondent did not have sufficient familiarity with that aspect of the instrument to comment and were not analysed further. This resulted in substantial variation in the number of responses between different instruments and to a lesser degree between different questions for the same instrument. Free text responses were considered thematically to provide further context to the quantitative analysis.

## 3 RESULTS

### 3.1 Sample description

There were 229 complete individual responses to the survey (see Table 2). The majority of respondents (77%, *n* = 177) were from England, with a further 20% (*n* = 46) coming from other UK nations. Despite the survey being advertised through UK channels, six respondents were from other countries (Australia, New Zealand and the Republic of Ireland). Psychiatrists made up the single largest group of respondents (36%, *n = *83), followed by nurses (24%, *n* = 55) and psychologists (22%, *n* = 50). Respondents choosing the response of ‘other’ for their professional background listed their occupation as Mental Health Act administrator, arts therapist and counsellor. Due to the use of snowball sampling, it was not possible to accurately calculate an overall response rate, however the survey was distributed to 4450 members of the RCPsych Forensic Faculty. If all 83 psychiatrist respondents were recruited via this mechanism, this represents a response rate of 2%. The majority of respondents reported working in medium secure mental health services (*n* = 117, 51%); 95 (41%) worked in low secure services, and 14 (6%) in high secure mental health services. Twenty‐one (95) reported working in other settings, including universities, primary mental health services and a locked rehabilitation ward.

**TABLE 2:** Characteristics of 229 survey respondents

Geographical area	*N*	%
East of England	15	7
London	12	5
Midlands	34	15
North East and Yorkshire	20	9
North West England	20	9
South West England	39	17
South East England	37	16
Scotland	26	11
Wales	16	7
Northern Ireland	4	2
Other	6	3

Professional background	*N*	%
Nursing	55	24
Psychiatry	83	36
Psychology	50	22
Occupational therapy	27	12
Social work	7	3
Other	7	3

Areas of work^a^	*N*	%(*N*/229)
High security forensic mental health service	14	6
Medium security forensic mental health service	117	51
Low security forensic mental health service	95	41
Community forensic mental health services	45	20
Prison mental health team	21	9
Other	21	9

^a^ Totals add up to more than 100% as respondents could indicate more than one area of work.

### 3.2 Familiarity with outcome measures and training in their use

Almost all respondents had heard of at least one instrument, with only 3 (1%) stating they had never heard of HCR‐20 and 19 (8%) that they had not heard of HoNOS‐Secure. The most familiar measure was the HCR‐20, with 75% (*n* = 171) of respondents stating that they used it regularly. The BEST Index was the least well‐known measure, with 75% (*n* = 172) of respondents stating they had not heard of it, and only three people reporting regular use (see Table 3).

**TABLE 3:** Respondents' familiarity with the selected instruments

	Regular use		Some experience		Heard of it, but not used it		Have not heard of it	
	*N*	%	*N*	%	*N*	%	*N*	%
HCR‐20	171	75	45	20	10	4	3	1
START	43	19	55	24	77	34	54	24
CANFOR	4	2	44	19	83	36	98	43
DUNDRUM	30	13	34	15	85	37	80	35
HoNOS‐Secure	104	45	56	24	50	22	19	8
LS/CMI	2	1	11	5	56	24	160	70
VRS	9	4	33	14	103	45	84	37
SAPROF	41	18	60	26	88	38	40	17
SVR‐20	23	10	77	34	90	39	39	17
BEST Index	3	1	11	5	43	19	172	75

Abbreviations: BEST, Behavioural Status; CANFOR, Camberwell Assessment of Need—Forensic; DUNDRUM, Dangerousness, Understanding, Recovery and Urgency Manual; HCR‐20, The Historical Clinical Risk 20; HoNOS‐S, Health of the Nation Outcome Scale Secure; LS/CMI, Level of Service Case Management Inventory; SVR‐20, Sexual Violence Risk 20; START, Short Term Assessment of Risk and Treatability; SAPROF, Structured Assessment of Protective Factors for risk of violence; VRS, Violence Risk Scale.

Respondents were not asked any further questions about an instrument if they indicated that they were not familiar with it. Furthermore, responses indicating ‘don't know’ to any subsequent question were excluded from the analysis. The results to subsequent questions are presented with the denominator as the number of respondents who were familiar with that instrument and were able to select an answer on the five‐point Likert scale of agreement. The denominator therefore varies considerably between instruments and between questions.

Respondents were most likely to report having been trained for using the HCR‐20 (81% of those familiar with it, *n* = 182). The LS/CMI had the lowest proportion of respondents expressing some familiarity with it having received training, with only two people saying that they had received formal training in its use and 11 (16%) reporting informal training (see Table 4)

**TABLE 4:** Respondents' level of training for the selected instruments

	Formal		Informal		None		Total *N*
	*N*	%	*N*	%	*N*	%	
HCR‐20	182	81	24	11	20	9	226
START	50	29	42	24	83	47	175
CANFOR	13	10	25	19	93	71	131
DUNDRUM	32	21	34	23	83	56	149
HoNOS‐Secure	61	29	77	37	72	34	210
LS/CMI	2	3	11	16	56	81	69
VRS	13	9	16	11	116	80	145
SAPROF	69	37	34	18	86	46	189
SVR‐20	41	22	29	15	120	63	190
BEST Index	3	5	8	14	46	81	57

Abbreviations: BEST, Behavioural Status; CANFOR, Camberwell Assessment of Need—Forensic; DUNDRUM, Dangerousness, Understanding, Recovery and Urgency Manual; HCR‐20, The Historical Clinical Risk 20; HoNOS‐S, Health of the Nation Outcome Scale Secure; LS/CMI, Level of Service Case Management Inventory; SVR‐20, Sexual Violence Risk 20; START, Short Term Assessment of Risk and Treatability; SAPROF, Structured Assessment of Protective Factors for risk of violence; VRS, Violence Risk Scale.

### 3.3 Comprehensiveness

Of the 10 tools, although the HCR‐20 had the highest proportion of respondents who viewed it as comprehensive, 39% (*n* = 85) of respondents familiar with it agreed or strongly agreed that it is comprehensive. The LS/CMI had the lowest proportion who viewed it as comprehensive, with 58% (n = 18) of those familiar with it at the opposite pole, either disagreeing or strongly disagreeing that it is comprehensive (see Table 5).

**TABLE 5:** Content validity and practical applicability of the selected instruments according to respondents

	Strongly agree		Agree		Neither agree nor Disagree		Disagree		Strongly disagree		Total
	*N*	%	*N*	%	*N*	%	*N*	%	*N*	%	*N*
Comprehensive											
HCR‐20	43	20	42	19	36	16	65	30	33	15	219
START	11	9	27	23	28	24	33	27	21	17	120
CANFOR	2	3	11	16	23	34	21	31	11	16	68
DUNDRUM	8	8	20	21	28	29	31	32	10	10	97
HoNOS‐Secure	15	9	26	15	45	26	56	32	31	18	173
LS/CMI	0	0	2	6	11	36	9	29	9	29	31
VRS	8	10	9	11	24	30	23	29	16	20	80
SAPROF	11	9	24	19	39	30	38	29	17	13	129
SVR‐20	13	10	14	11	35	28	45	36	19	15	126
BEST Index	1	3	2	7	15	52	5	17	6	21	29
Easy to use											
HCR‐20	22	10	61	28	41	19	70	32	24	11	218
START	15	14	50	45	27	24	13	12	6	5	111
CANFOR	2	3	20	36	19	34	11	20	4	7	56
DUNDRUM	10	13	29	38	22	29	8	10	8	10	77
HoNOS‐Secure	28	17	87	52	34	20	11	7	6	4	166
LS/CMI	0	0	7	31	12	52	1	4	3	13	23
VRS	2	4	13	24	20	38	14	26	4	8	53
SAPROF	12	11	49	43	32	28	14	12	7	6	114
SVR‐20	6	6	33	31	20	19	36	34	10	10	105
BEST Index	1	5	3	14	10	48	5	24	2	9	21
Relevant											
HCR‐20	95	43	78	35	22	10	20	9	8	4	223
START	25	20	43	34	36	29	9	7	12	10	125
CANFOR	5	8	15	23	31	48	7	11	7	11	65
DUNDRUM	23	26	33	37	26	29	5	6	3	3	90
HoNOS‐Secure	27	15	59	33	44	24	29	16	21	12	180
LS/CMI	0	0	9	33	10	37	3	11	5	19	27
VRS	17	25	19	28	20	30	3	4	8	12	67
SAPROF	33	26	55	43	26	20	5	4	8	6	127
SVR‐20	35	28	45	36	26	21	13	10	7	6	126
BEST Index	2	7	9	31	13	45	3	10	2	7	29
Progress											
HCR‐20	52	24	101	46	32	15	25	11	10	5	220
START	23	19	50	42	28	24	12	10	6	5	119
CANFOR	2	3	20	33	29	48	6	10	3	5	60
DUNDRUM	14	17	33	41	21	26	11	14	2	2	81
HoNOS‐Secure	20	12	58	34	48	28	23	14	21	12	170
LS/CMI	0	0	2	7	20	71	4	14	2	7	28
VRS	9	14	21	32	21	32	10	15	4	6	65
SAPROF	17	15	55	49	28	25	9	8	4	4	113
SVR‐20	17	15	51	46	29	26	13	12	2	2	112
BEST Index	1	4	5	21	14	59	2	8	2	8	24
Care planning											
HCR‐20	79	36	99	45	25	11	11	5	6	3	220
START	29	26	44	39	23	20	12	11	5	4	113
CANFOR	3	5	25	45	21	38	4	7	3	5	56
DUNDRUM	10	12	26	32	27	33	14	17	4	5	81
HoNOS‐Secure	16	10	28	17	56	34	36	22	30	18	166
LS/CMI	2	8	6	25	7	29	7	29	2	8	24
VRS	11	17	25	38	14	22	10	15	5	8	65
SAPROF	37	31	53	45	16	14	7	6	5	4	118
SVR‐20	27	24	56	49	17	15	10	9	4	4	114
BEST Index	2	9	5	24	11	53	2	10	1	5	21

Abbreviations: BEST, Behavioural Status; CANFOR, Camberwell Assessment of Need—Forensic; DUNDRUM, Dangerousness, Understanding, Recovery and Urgency Manual; HCR‐20, The Historical Clinical Risk 20; HoNOS‐S, Health of the Nation Outcome Scale Secure; LS/CMI, Level of Service Case Management Inventory; SVR‐20, Sexual Violence Risk 20; START, Short Term Assessment of Risk and Treatability; SAPROF, Structured Assessment of Protective Factors for risk of violence; VRS, Violence Risk Scale.

Comments from respondents included concerns about the focus on assessing risk of harm to others in many instruments, with limited attention paid to actual outcomes. They also highlighted the importance of selecting a measure that fits the characteristics of the particular population assessed and noted that a combination of measures might be necessary to cover all relevant outcomes.

#### 3.3.1 Ease of use

The HoNOS‐Secure had the highest proportion of respondents viewing it as easy to use with 69% (*n* = 83) of those familiar with it either agreeing or strongly agreeing. The SVR‐20 and HCR‐20 had the lowest proportions of respondents familiar with the instruments stating that they are easy to use and also the highest proportions disagreeing/strongly disagreeing that they are easy (n = 46, 44%; n = 94, 43% respectively) (see Table 5).

Some free text responses presented the view that completing instruments was often a purely administrative task of no clinical value, while others emphasised the need for a balance between ease of use and meaningfulness. Poor IT was emphasised as a barrier to completing measures, while training, team familiarity and access to manuals were enablers.

### 3.4 Relevance

The HCR‐20 had the highest proportion of respondents familiar with it who felt that it is relevant with 78% (*n* = 173) agreeing or strongly agreeing. The LS/CMI had the lowest proportion of respondents who viewed it as relevant with 30% (*n* = 8) of those familiar with it disagreeing or strongly disagreeing (see Table 5).

In free text comments, respondents highlighted the importance of selecting a measure that fits the characteristics of the population being assessed. Several respondents also raised concerns that measures designed to assess risk or needs are not relevant to measure outcomes.

### 3.5 Applicability of outcome measures in clinical practice

The HCR‐20 had the highest proportion of respondents familiar with it, with 70% (*n* = 153) either agreeing or strongly agreeing, while the HoNOS‐Secure was least likely to be viewed this way, with just over one quarter of respondents familiar with it (n = 44, 26%) disagreeing or strongly disagreeing that it has value in this particular way. The HCR‐20 was also viewed as the most relevant for care planning with 81% (*n* = 178) of those familiar with it either agreeing or strongly agreeing, whilst, by contrast, the HoNOS‐Secure had the lowest proportion of respondents familiar with it (n = 66, 40%) either disagreeing or strongly disagreeing that it was good for care planning (see Table 5).

Free text responses highlighted that measures were only helpful if used in an appropriate population. Some respondents commented that those measures focusing on skills and strengths were most useful, while others noted that risk assessments could be helpful to measure insight. Comments noted that the measures can be useful to identify targets for interventions for individual patients. Others highlighted that measures can be useful to help patients take ownership of risk, but should be collaborative.

#### 3.5.1 Patient involvement

The CANFOR and BEST had the highest proportion of respondents familiar with the instruments stating that patients were fully involved in their rating, although the number of responses was very small (both 22%, *n* = 8 and *n = *2, respectively). Of the respondents familiar with the HCR‐20, 55% (*n* = 113) indicated that patients were at least partially involved. This percentage rose to 62% of respondents indicating that patients were partially involved with rating the SAPROF, although the numbers were smaller (*n* = 56). The DUNDRUM and HoNOS‐Secure had the highest proportion of responses stating that patients were not involved at all (81%, *n* = 53 and 79%, *n* = 113 respectively) (see Table 6).

**TABLE 6:** Patient involvement in rating of the selected instruments according to respondents

	Fully involved		Partially involved		Informed, but not involved		Not involved or informed		Total
	*N*	%	*N*	%	*N*	%	*N*	%	*N*
HCR‐20	29	14	84	41	47	23	47	23	207
START	15	16	31	32	22	23	28	29	96
CANFOR	8	22	9	25	10	28	9	25	36
DUNDRUM	2	3	11	17	11	17	42	64	66
HoNOS‐Secure	12	9	17	12	33	23	80	56	142
LS/CMI	1	9	4	37	3	27	3	27	11
VRS	3	9	8	25	9	28	12	38	32
SAPROF	16	18	40	44	16	18	19	21	91
SVR‐20	7	9	25	32	20	26	26	33	78
BEST Index	2	22	4	45	1	11	2	22	9

Abbreviations: BEST, Behavioural Status; CANFOR, Camberwell Assessment of Need—Forensic; DUNDRUM, Dangerousness, Understanding, Recovery and Urgency Manual; HCR‐20, The Historical Clinical Risk 20; HoNOS‐S, Health of the Nation Outcome Scale Secure; LS/CMI, Level of Service Case Management Inventory; SVR‐20, Sexual Violence Risk 20; START, Short Term Assessment of Risk and Treatability; SAPROF, Structured Assessment of Protective Factors for risk of violence; VRS, Violence Risk Scale.

In the free text comments, some respondents stated that patients did not like being involved in the rating process, especially measures that are long or complicated and that some patients could find the process of rating distressing, in particular items related to risk. Other responses mentioned the variability in patient involvement, depending on patient preference and on the patient's state of mind at the time of rating.

## 4 DISCUSSION

### 4.1 Variable familiarity and training with instruments

In this survey of 229 clinicians on 10 of the most common instruments used to track the outcomes of care and treatment in forensic mental health services, we found wide variation in respondents' familiarity with the instruments. This pattern does not fully mirror the frequency with which these instruments appear in the literature. The HCR‐20 was the most widely known, followed closely by the HoNOS‐Secure. This may be partly explained by the fact that these are mandated in the countries where most respondents were based (NHS England, 2020). Many instruments were only known by a small minority of respondents. Reported training also varied in a way that may reflect the requirements of individual instruments; the HCR‐20, for example, mandates extensive training before use, while there is no training requirement for the HoNOS‐Secure (Teo et al., 2012).

### 4.2 The importance of content validity

Given the widespread familiarity with, and use of, at least some of the 10 instruments, particularly HCR‐20 and HONOS‐Secure, it is important that such instruments are fit for purpose, and that clinicians are confident in their usefulness and validity. Services using these instruments should pay careful attention to the way that they are being implemented and interpreted, to ensure that the intended use is supported by evidence for the relevant psychometric properties (Prinsen et al., 2018). In particular, if instruments are being used as de facto outcome measures, then it is essential that such measures have the requisite content validity for all relevant stakeholders (Terwee et al., 2018).

None of the selected instruments appear to cover all outcomes of interest to clinicians. Clinicians and patients, in conjunction with researchers, are best placed to consider what further instruments may be required and what may be the best combination of instruments, to then inform policy makers from the evidence (NHS England and NHS Improvement, 2016). Respondents highlighted the need to use repeated measures to track progress over time (Longdon et al., 2018). To do this effectively requires instruments that are both comprehensive and ease to use, with adequate dynamic components. However, many of these instruments have a large proportion of static items, based on historical risk factors, which are not responsive to change.

### 4.3 Practical application of outcome measures

It is important to minimise the administrative burden on clinical staff by making instruments quick to complete, while also asking the right questions to add meaningfully to patient care. As there is a balance between the time taken to complete these instruments and their perceived usefulness, attention should be paid to whether any additional complexity is worthwhile. Efforts should also be made to mitigate the impact on clinicians' workloads, for example, by incorporating instruments in to the electronic patient record (Kilbourne, Keyser, & Pincus, 2010).

There is a need for careful interpretation of instruments when formulating care plans. These should support good clinical practice, rather than replace it, even when patients are contributing directly to ratings. Care plans based on standardised instruments risk being generic without adequate input from the patient (Lovell et al., 2019). The lack of patient‐reported scales means that any care plans based on these scales may not be sufficiently collaborative (Coffey et al., 2019). Measures need to be simple and accessible for use by all relevant raters, including clinicians and patients (Keetharuth et al., 2018).

#### 4.3.1 Maximising patient involvement

Patients' involvement with the rating process of the selected instruments was mixed. The CANFOR, which is the only instrument which was originally developed to include a patient‐reported scale, performed reasonably well in this respect, with 47% of respondents familiar with it reporting at least partial patient involvement (Thomas et al., 2008). The proportion of respondents stating that patients were fully involved in rating the DUNDRUM was the lowest for any instrument, despite this also having a patient‐rated scale (Davoren et al., 2015). This may be because the patient‐rated scale was developed after the clinician one and therefore clinicians may not be so familiar with it. The CANFOR was outperformed in having at least partial involvement by the HCR‐20, SAPROF and BEST scales, for all of which the majority of respondents reported full or partial patient involvement. While these figures may appear encouraging on the surface, a formal patient‐rating element is required to ensure that this part of the assessment is done. Such dedicated patient‐reported scales may capture nuances of patients' own perspectives, which may otherwise be lost by simply involving them with rating a scale designed for clinicians.

While patient involvement in measuring outcomes is recommended, it is essential that the measures used are appropriate for this purpose. Patients should be involved throughout the development of instruments used to measure their outcomes. Authors may want to consider how patients are involved in future iterations of their existing instruments (Grundy et al., 2019). Further research is required to understand better the barriers and facilitators to patient involvement in outcome measure development and rating in forensic services (Spaulding et al., 2019).

### 4.4 Strengths and limitations

While use of survey methodology enabled the participation of professionals from a wide range of backgrounds, services and geographical areas within the UK, it was not possible to calculate an overall response rate due to the way that the survey link was distributed. The estimated participation rate by forensic psychiatrists is only 2%, so the views expressed may not be representative of the wider body of forensic psychiatrists. Although we can say that the proportionate geographic distribution of responders is more‐or‐less in line with service distribution, it is harder to estimate professional distribution. Unlike the list of forensic psychiatrists kept by the RCPsych, there are not similar records of the numbers working in other professional categories or comprehensive networks for distributing an invitation to participate in a survey. The use of professional networks may have created a response bias, as those in contact with such networks may be more engaged with managerial issues, such as the measurement of outcomes (Floyd & Fowler, 2013). Despite efforts to involve other professional groups through multi‐disciplinary networks, psychiatrists formed the largest single group of respondents. This may have skewed the responses towards the concerns of this one professional group. Although 229 individual respondents completed the survey overall, the numbers who were able to comment on all the different aspects of the various selected instruments varied widely. This meant that the number of responses for some of the less well‐known instruments could be very small.

The survey relied on respondents to judge whether they were sufficiently familiar with an instrument to form an opinion about each aspect. The opinions on instruments that respondents had not personally used could therefore have been based on limited familiarity. Although free text responses allowed views to be collected beyond the limits of the quantitative questions, the survey format did not allow for probing to explore the views of respondents in more detail, which would have been possible with an interview or focus group (Green & Thorogood, 2018). The study was limited to 10 common instruments, whereas there are many other instruments available to forensic mental health services. Respondents were all clinicians, meaning that the views of patients and other key stakeholders, particularly patients, were not considered. Although a dedicated patient advisory group were consulted by the research team on a wider project to investigate outcome measurement in forensic mental health services, of which this survey was a part, they were not involved directly in the design of the survey itself.

## 5 CONCLUSIONS

Almost all of the participating 229 clinicians were familiar with some of the 10 measures we selected for consideration based on the supporting empirical literature. While the respondents constituted only a small proportion of all practising forensic psychiatrists in the UK, they were representative of the country geographically and, as clearly interested in this field, have an important perspective on outcome measurement. Only one of the 10 instruments studied (HoNOS‐Secure) was primarily designed as an outcome measure and yet these clinicians were least likely to rate it as useful in this respect. Patients have not been much involved in the design of these scales. At best, clinicians reported their full involvement in under 25% of ratings in practice, although more than half reported at least partial patient involvement with some of the more commonly used instruments, such as the HCR‐20. It was notable that for two of the scales, including the commonly used HoNOS Secure, it was reported that more than half the patients were not informed that the ratings were being made. Future research in this area should focus on co‐production of tools that support the reliable and valid measurement of change in areas of mental health, behaviour and need considered integral in a pathway to recovery and safety.

## CONFLICT OF INTEREST

The authors report no conflict of interests.

## Supporting information

Supporting Information S1

## Data Availability

The data that support the findings of this study are available from the corresponding author upon reasonable request.
