# Supplementary material for: Ten outcome measures in forensic mental health: A survey of clinician views on comprehensiveness, ease of use and relevance
Source: Crim Behav Ment Health. 2021 Nov 9;31(6):372–86. doi: 10.1002/cbm.2221 (PMC9299034; doi:10.1002/cbm.2221)
Supplement: Supplementary file 1 — Supporting Information S1 [file CBM-31-372-s001.docx]

**Appendix**

Table A1

*Patient involvement in rating of the selected instruments according to respondents*

|  | Fully involved | | Partially involved | | Informed, but not involved | | Not involved or informed | | Total |
| --- | --- | --- | --- | --- | --- | --- | --- | --- | --- |
|  | N | % | N | % | N | % | N | % | N |
| HCR-20 | 29 | 14 | 84 | 41 | 47 | 23 | 47 | 23 | 207 |
| START | 15 | 16 | 31 | 32 | 22 | 23 | 28 | 29 | 96 |
| CANFOR | 8 | 22 | 9 | 25 | 10 | 28 | 9 | 25 | 36 |
| DUNDRUM | 2 | 3 | 11 | 17 | 11 | 17 | 42 | 64 | 66 |
| HoNOS-S | 12 | 9 | 17 | 12 | 33 | 23 | 80 | 56 | 142 |
| LS/CMI | 1 | 9 | 4 | 37 | 3 | 27 | 3 | 27 | 11 |
| VRS | 3 | 9 | 8 | 25 | 9 | 28 | 12 | 38 | 32 |
| SAPROF | 16 | 18 | 40 | 44 | 16 | 18 | 19 | 21 | 91 |
| SVR-20 | 7 | 9 | 25 | 32 | 20 | 26 | 26 | 33 | 78 |
| BEST | 2 | 22 | 4 | 45 | 1 | 11 | 2 | 22 | 9 |

Table A2

*Perceived relevance of the selected instruments to forensic services and their use as outcome measures in clinical practice according to respondents*

|  | Strongly agree | | Agree | | Neither agree nor disagree | | Disagree | | Strongly disagree | | Total |
| --- | --- | --- | --- | --- | --- | --- | --- | --- | --- | --- | --- |
|  | N | % | N | % | N | % | N | % | N | % | N |
| **Relevant** |  |  |  |  |  |  |  |  |  |  |  |
| HCR-20 | 95 | 43 | 78 | 35 | 22 | 10 | 20 | 9 | 8 | 4 | 223 |
| START | 25 | 20 | 43 | 34 | 36 | 29 | 9 | 7 | 12 | 10 | 125 |
| CANFOR | 5 | 8 | 15 | 23 | 31 | 48 | 7 | 11 | 7 | 11 | 65 |
| DUNDRUM | 23 | 26 | 33 | 37 | 26 | 29 | 5 | 6 | 3 | 3 | 90 |
| HoNOS-S | 27 | 15 | 59 | 33 | 44 | 24 | 29 | 16 | 21 | 12 | 180 |
| LS/CMI | 0 | 0 | 9 | 33 | 10 | 37 | 3 | 11 | 5 | 19 | 27 |
| VRS | 17 | 25 | 19 | 28 | 20 | 30 | 3 | 4 | 8 | 12 | 67 |
| SAPROF | 33 | 26 | 55 | 43 | 26 | 20 | 5 | 4 | 8 | 6 | 127 |
| SVR-20 | 35 | 28 | 45 | 36 | 26 | 21 | 13 | 10 | 7 | 6 | 126 |
| BEST | 2 | 7 | 9 | 31 | 13 | 45 | 3 | 10 | 2 | 7 | 29 |
|  |  |  |  |  |  |  |  |  |  |  |  |
| **Progress** |  |  |  |  |  |  |  |  |  |  |  |
| HCR-20 | 52 | 24 | 101 | 46 | 32 | 15 | 25 | 11 | 10 | 5 | 220 |
| START | 23 | 19 | 50 | 42 | 28 | 24 | 12 | 10 | 6 | 5 | 119 |
| CANFOR | 2 | 3 | 20 | 33 | 29 | 48 | 6 | 10 | 3 | 5 | 60 |
| DUNDRUM | 14 | 17 | 33 | 41 | 21 | 26 | 11 | 14 | 2 | 2 | 81 |
| HoNOS-S | 20 | 12 | 58 | 34 | 48 | 28 | 23 | 14 | 21 | 12 | 170 |
| LS/CMI | 0 | 0 | 2 | 7 | 20 | 71 | 4 | 14 | 2 | 7 | 28 |
| VRS | 9 | 14 | 21 | 32 | 21 | 32 | 10 | 15 | 4 | 6 | 65 |
| SAPROF | 17 | 15 | 55 | 49 | 28 | 25 | 9 | 8 | 4 | 4 | 113 |
| SVR-20 | 17 | 15 | 51 | 46 | 29 | 26 | 13 | 12 | 2 | 2 | 112 |
| BEST | 1 | 4 | 5 | 21 | 14 | 59 | 2 | 8 | 2 | 8 | 24 |
|  |  |  |  |  |  |  |  |  |  |  |  |
| **Care planning** |  |  |  |  |  |  |  |  |  |  |  |
| HCR-20 | 79 | 36 | 99 | 45 | 25 | 11 | 11 | 5 | 6 | 3 | 220 |
| START | 29 | 26 | 44 | 39 | 23 | 20 | 12 | 11 | 5 | 4 | 113 |
| CANFOR | 3 | 5 | 25 | 45 | 21 | 38 | 4 | 7 | 3 | 5 | 56 |
| DUNDRUM | 10 | 12 | 26 | 32 | 27 | 33 | 14 | 17 | 4 | 5 | 81 |
| HoNOS-S | 16 | 10 | 28 | 17 | 56 | 34 | 36 | 22 | 30 | 18 | 166 |
| LS/CMI | 2 | 8 | 6 | 25 | 7 | 29 | 7 | 29 | 2 | 8 | 24 |
| VRS | 11 | 17 | 25 | 38 | 14 | 22 | 10 | 15 | 5 | 8 | 65 |
| SAPROF | 37 | 31 | 53 | 45 | 16 | 14 | 7 | 6 | 5 | 4 | 118 |
| SVR-20 | 27 | 24 | 56 | 49 | 17 | 15 | 10 | 9 | 4 | 4 | 114 |
| BEST | 2 | 9 | 5 | 24 | 11 | 53 | 2 | 10 | 1 | 5 | 21 |
